# Pharmacogenomics of Cytochrome P450 3A4: Recent Progress Toward the “Missing Heritability” Problem

**DOI:** 10.3389/fgene.2013.00012

**Published:** 2013-02-25

**Authors:** Kathrin Klein, Ulrich M. Zanger

**Affiliations:** ^1^Dr. Margarete Fischer Bosch Institute of Clinical Pharmacology, StuttgartStuttgart, Germany; ^2^University of TübingenTübingen, Germany

**Keywords:** cytochrome P450, CYP3A4, pharmacogenomics, pharmacogenetics, drug metabolism, heritability

## Abstract

CYP3A4 is the most important drug metabolizing enzyme in adult humans because of its prominent expression in liver and gut and because of its broad substrate specificity, which includes drugs from most therapeutic categories and many endogenous substances. Expression and function of CYP3A4 vary extensively both intra- and interindividually thus contributing to unpredictable drug response and toxicity. A multitude of environmental, genetic, and physiological factors are known to influence CYP3A4 expression and activity. Among the best predictable sources of variation are drug–drug interactions, which are either caused by pregnane X-receptor (PXR), constitutive androstane receptor (CAR) mediated gene induction, or by inhibition through coadministered drugs or other chemicals, including also plant and food ingredients. Among physiological and pathophysiological factors are hormonal status, age, and gender, the latter of which was shown to result in higher levels in females compared to males, as well as inflammatory processes that downregulate CYP3A4 transcription. Despite the influence of these non-genetic factors, the genetic influence on CYP3A4 activity was estimated in previous twin studies and using information on repeated drug administration to account for 66% up to 88% of the interindividual variation. Although many single nucleotide polymorphisms (SNPs) within the *CYP3A* locus have been identified, genetic association studies have so far failed to explain a major part of the phenotypic variability. The term “missing heritability” has been used to denominate the gap between expected and known genetic contribution, e.g., for complex diseases, and is also used here in analogy. In this review we summarize *CYP3A4* pharmacogenetics/genomics from the early inheritance estimations up to the most recent genetic and clinical studies, including new findings about SNPs in *CYP3A4* (*22) and other genes (P450 oxidoreductase (POR), peroxisome proliferator-activated receptor alpha (PPARA)) with possible contribution to CYP3A4 variable expression.

## CYP3A4 Drug Substrates and Phenotyping

CYP3A4 is in the majority of individuals abundantly expressed in liver but population variability is extremely high (>100-fold). Average microsomal content has been estimated between ∼60 pmol/mg of microsomal protein (Ohtsuki et al., 2012), 110 pmol/mg (Klein et al., 2012), and ∼150 pmol/mg (Westlind-Johnsson et al., 2003). Hepatic expression of the other CYP3A enzymes CYP3A5, CYP3A7, and CYP3A43 is much lower in adult Caucasians, although CYP3A5 may contribute up to about 50% of the CYP3A pool in individuals with at least one CYP3A5*1 allele and with low CYP3A4 expression (Hustert et al., 2001a; Kuehl et al., 2001; Koch et al., 2002; King et al., 2003; Klees et al., 2005; McCune et al., 2005). The role of the minor CYP3A forms has been reviewed by others (Burk and Wojnowski, 2004; Daly, 2006). CYP3A4 is thus one of the most abundantly expressed cytochromes in human liver contributing on average about 15–30% to the microsomal P450 pool. Therefore and owing to their extraordinarily broad substrate selectivity CYP3A enzymes play a major role in the metabolism of ∼30–40% of all clinically used drugs (Evans and Relling, 1999; Zanger et al., 2008). These include preferentially lipophilic and bulky compounds from almost all therapeutic categories, including tacrolimus, cyclosporin A, erythromycin, ifosfamide, tamoxifen, benzodiazepines, several statins, antidepressants, opioids, and many more, as summarized in several previous reviews (Bu, 2006; Liu et al., 2007; Zanger et al., 2008). As CYP3A4 is also an efficient steroid hydroxylase, it has in addition an important role in the catabolism of several endogenous steroids like testosterone, progesterone, cortisol, and bile acids (Patki et al., 2003; Bodin et al., 2005).

The high sequence similarity of >85% between the CYP3A isozymes CYP3A4 and CYP3A5 leads to highly similar substrate selectivity between these isoforms (Williams et al., 2002). CYP3A4 and CYP3A5 activities are therefore not specifically discriminable although some probe drugs showed higher selectivity for CYP3A4-including, for example, erythromycin *N*-demethylation (Wrighton et al., 1990) and atorvastatin ortho-hydroxylation (Feidt et al., 2010). Several *in vivo* test substrates are available and have been compared to each other, e.g., midazolam, erythromycin, quinine, and nifedipine (Liu et al., 2007). Endogenous marker activities have also been proposed as functional CYP3A-markers, including the 6β-hydroxylation of cortisol and the 4β-hydroxylation of cholesterol, but some limitations due to considerable intraindividual variability exist (Chen et al., 2006; Fuhr et al., 2007). Remarkably, however, data obtained with different CYP3A substrates or endogenous markers are not generally well correlated to each other and have therefore to be selected carefully with respect to the expected change in activity and design of the study (Galteau and Shamsa, 2003; Chen et al., 2006; Fuhr et al., 2007). Functional CYP3A phenotyping therefore continues to be a non-trivial problem.

## Degree of Heritability

CYP3A4 drug oxidation phenotypes are highly variable but unimodally distributed. Nevertheless there is indication of substantial heritability. For example, antipyrine 4-hydroxylation rate, which is mainly catalyzed by CYP3A4 (Engel et al., 1996), was reported to be largely inherited (85%) as shown in early twin studies (Penno et al., 1981). Moreover, a high degree of heritability for CYP3A4 drug oxidation capacity toward several of its substrates (erythromycin: 89% and midazolam: 96%) was estimated by a repeated drug administration approach (Ozdemir et al., 2000). A recent study used a classical twin model approach in combination with a St. John’s Wort induction regimen and quinine sulfate metabolism as CYP3A4 activity measure to elucidate genetic versus non-genetic contribution to variable CYP3A4 induction. Although the uninduced levels were not recorded, the induced CYP3A4 activity heritability was estimated to be at least 66% and environmental factors like BMI, alcohol use, and smoking habit/quantity contributed at least 20% to the variability (Rahmioglu et al., 2011).

In contrast to the genetically polymorphic *CYP2D6*, which is mostly determined genetically with only minor contribution by environmental factors, most of the previously studied *CYP3A4* polymorphisms are either rare or lack phenotypic effect and are thus unable to explain a sizeable fraction of heritable variation. The first documented *CYP3A4* polymorphism is the frequently studied proximal promoter variant CYP3A4*1B (−392A > G, rs2740574) which occurs in white populations at ∼2–9% but at higher frequencies in Africans. This single nucleotide polymorphism (SNP) was initially found to be associated with higher tumor grade and stage in prostate cancer and showed higher nifedipine oxidase activity in human livers (Rebbeck et al., 1998). Association of CYP3A4*1B with markers of advanced disease was confirmed by some but not all further studies (Keshava et al., 2004; Perera et al., 2009). Although one *in vitro* study on the promoter variant found higher transcriptional activity in cell culture experiments using luciferase constructs, as well as changed protein binding by electrophoretic mobility shift assay (Amirimani et al., 2003), the functional effect of this variant remains questionable given the controversial data reported in several other *in vitro* and human liver studies (Wandel et al., 2000; Spurdle et al., 2002; Rodríguez-Antona et al., 2005; Klein et al., 2012). Also, a study with healthy volunteers phenotyped using the dextromethorphan/methoxymorphinan ratio failed to find an association of CYP3A4*1B or *2 with CYP3A4 activity (García-Martín et al., 2002). Recently, resequencing and haplotype tagging studies have been carried out at the *CYP3A* locus in ethnically diverse populations (Thompson et al., 2006; Schirmer et al., 2007; Perera et al., 2009; Perera, 2010). These studies addressed the problem of “missing heritability” of CYP3A4 phenotype variability by considering haplotype structure at the *CYP3A* locus, which was found to be of some relevance. A markedly increased occurrence of rare variants and the presence of a homogeneous group of long-range haplotypes at high frequency were observed in non-African populations (Thompson et al., 2004). Because of their involvement in the metabolism not only of naturally occurring foreign compounds, such as flavonoids, diterpenoids, and other herbal constituents (Zhou et al., 2007) but also of endogenous chemicals such as uroporphyrin (Franklin et al., 2000) it has been suggested that molecular adaptation to the changing environment may have occurred for genes at the *CYP3A* locus. Thus, it has been postulated that a region from about 40 kb upstream of the *CYP3A4* gene promoter to intron 6 was under positive selection in humans (Thompson et al., 2004; Chen et al., 2009). In addition, despite the controversy about the CYP3A4*1B allele, a “high haplotype homozygosity” in European Caucasians in contrast to African Americans was observed, which may indicate a negative selection pressure that eliminated the *1B allele in non-African populations (Schirmer et al., 2006). It should also be mentioned that interaction between SNP/haplotype and gender have been reported to impact on gene expression, further complicating the situation (Schirmer et al., 2007).

An intronic variant rs4646450, located in the *CYP3A5* gene, has recently been associated with reduced tacrolimus dosage requirement in Japanese patients (Onizuka et al., 2011), with reduced endogenous serum dehydroepiandrosterone sulfate concentrations in over 14,000 individuals (Zhai et al., 2011) and was also associated with decreased protein expression and activity of CYP3A4 in human liver, explaining about 3–5% of hepatic variability (Klein et al., 2012). These data underline the importance of haplotype structure throughout the *CYP3A* locus, and they raise questions about causality of the various intronic and regulatory variants. It has been suggested generally that the most penetrant risk alleles may be of negative selective pressure and therefore be rare (Sadee, 2012). This may have led to their oversight in small sized screening studies or due to gene-environment interactions which can substantially mask genetic effects (Rahmioglu et al., 2011; Klein et al., 2012; Sadee, 2012).

Recently, however, an intron 6 variant rs35599367C > T (CYP3A4*22) was identified by a systematic screen for SNPs showing allelic mRNA expression imbalance in human liver (Wang et al., 2011). The effect of the variant was not confounded by sex or other variables and it accounted for 7% of the mRNA expression variability in a cohort of 93 liver samples. Expression in cultured cells transfected with minigenes containing exon 6, intron 6, and exon 7 reproduced the functional effect of the T-allele to decrease mRNA *in vitro*, suggesting that it could be the causal variant. As no associated splice variants or other hints to the exact mechanism were found, the variant was supposed to affect nascent RNA elongation rate (Wang et al., 2011; Sadee, 2012). In another recent study using 150 liver samples this variant was associated with decreased protein levels, but not when corrections were made for multiple testing (Klein et al., 2012).

Furthermore, the potential impact of CYP3A4 protein variants on drug–drug interaction was emphasized in a recent *in vitro* study using recombinantly expressed variant CYP3A4 proteins 3A4.2, 3A4.7, 3A4.16, and 3A4.18, where inhibitory profile differences of itraconazole and cimetidine were described in relation to the CYP3A4 variants (Akiyoshi et al., 2011). Thus, although most of the protein variants are too rare to make an impact on a population scale, individual carriers of such variants may have risk not only for impaired drug metabolism but also for a different drug–drug interaction profile.

## CYP3A4 Polymorphisms – Clinical Impact

Most previous clinical studies that implemented *CYP3A* genotyping are not considered here because they used mainly the CYP3A5*3 polymorphism as marker. The reader is referred to other reviews on this topic (Burk and Wojnowski, 2004; Daly, 2006). Table 1 shows a selection of *CYP3A4*-including pharmacogenetic *in vivo* studies. Although the effects of the novel intron 6 variant were not very pronounced *in vitro*, its impact became more apparent by *in vivo* pharmacokinetic and pharmacodynamic studies. Significant association of CYP3A4*22 with decreased AUC_0−∞_ ratio in atorvastatin-treated volunteers or 1.7- to 5-fold reduced statin dose of T-allele carriers compared to non-T carriers to achieve optimal lipid control was shown in volunteer and clinical studies (Wang et al., 2011; Klein et al., 2012). The association of CYP3A4*22 with simvastatin lipid-lowering response was shown in another clinical study (Elens et al., 2011a). Furthermore, renal transplant recipients who were carriers of the low-expressor T-allele had a 33% reduced mean daily-dose requirement to reach the same tacrolimus blood concentration compared to homozygotes for the wild type allele (Elens et al., 2011b) and 1.6- to 2.0-fold higher dose-adjusted trough blood levels of tacrolimus and cyclosporine A in stable renal transplant patients (Elens et al., 2012), both indicating lower CYP3A4 activity. Despite these consistent reports, the rather low frequency of the intron 6 SNP [global minor allele frequency (MAF) 2.1%, Caucasians 3–8%] limits broader contribution to overall CYP3A4 variability.

**Table 1:** **Selected pharmacogenetic *in vivo* studies with CYP3A4-relevant data**.

	Substrate/metabolite	Study design	Subjects	Genotypes	Remarks/findings
Penno et al. (1981)	Antipyrine (orally)/4-OH-antipyrine	Twin study	Adult male unmedicated twins (10 unrelated, 10 monozygotic twins, 10 dizygotic twins)	No	Heritability 0.88
Ball et al. (1999)	Erythromycin, nifedipine	Five racial groups Hispanics, African American, Asian	802 Healthy volunteers	−292 (5′UTR) in *CYP3A4*	No effect on 3A4 dependent demethylation of erythromycin or nifedipine metabolism
Ozdemir et al. (2000)	10 Substrates	Repeated drug administration method	161 (Meta-analysis from 16 studies, literature search)	No	Genetic contribution to hepatic CYP3A4 activity 0.96–0.66
García-Martín et al. (2002)	Dextromethorphan/3′-methoxymorphinan ratio	Single oral dose with 24 h urine collection	76 Healthy volunteers (white subjects)	CYP3A4*1B, *2, *4, *5, *6, *8, *11, *12, *13	No association of *1B with activity phenotype
Floyd et al. (2003)	Midazolam oral/systemic clearance; erythromycin breath test	Constitutive and induced with rifampicin	57 Healthy subjects (European, African American)	CYP3A4*1B, CYP3A5*3, CYP3A5*6, and CYP3A5*7; MDR1 ex21/26	Fold increase after rifampicin is related to CYP3A4*1B
Hesselink et al. (2003)	Variability in cyclosporine and tacrolimus PK	Pharmacokinetic	110 + 64 Kidney transplant recipients	CYP3A4*1B, CYP3A5*3, MDR1_3435C > T	CYP3A4*1B: lower dose-adjusted trough tacrolimus levels
Hesselink et al. (2004)	Population PK of cyclosporine		151 Kidney and heart transplant recipients	CYP3A4*1B and *3, CYP3A5*3 and *6, MDR1_3435C > T	CYP3A4*1B: significantly higher oral cyclosporine clearance
He et al. (2005)	Midazolam oral clearance		26 Healthy volunteers, mixed ethnicity	Novel variants/haplotypes	CYP3A4*VI – CYP3A5*3A with only limited impact on CYP3A metabolism
Wang et al. (2005)	Lipid-lowering by simvastatin; 6β-OH-cortisol/cortisol		211 Hyperlipidemic Chinese patients	CYP3A4*4, *5, *6	CYP3A4*4 was related to a decrease of CYP3A4 activity, and seemed to increase the lipid-lowering effects of simvastatin
Diczfalusy et al. (2009)	4β-OH cholesterol/cholesterol	Rifampicin induction	24 Unrelated healthy volunteers	No	Plasma 4β-hydroxycholesterol has half-life of 17 days, low intraindividual variability
Diczfalusy et al. (2011)	4β-OH cholesterol/cholesterol, midazolam, quinine	Rifampicin induction; ritonavir inhibition	135 Tanzanian, 136 Swedes, 146 Korean	CYP3A5*1	4β-OH cholesterol/cholesterol is a good measure for long-term studies, midazolam/quinine rather for short term studies
Wang et al. (2011)	Required doses for optimal lipid control		235 Patients with stable doses of atorvastatin, simvastatin, or lovastatin	13 SNPs including CYP3A4*1B and *22	T-allele required significant lower statin doses for optimal lipid control
Chen et al. (2011)	Methadone and metabolite plasma concentrations and withdrawal scores		366 Han Chinese patients with heroin addiction under methadone maintenance treatment	*CYP3A4* rs4646440/rs2242480 and others	*CYP3A4* rs4646440, rs2242480 significantly associated with severity of withdrawal symptoms and side effects
Suhre et al. (2011)	Urine metabolic traits	GWAS KORA S4 population study	862 Independent male (Germany)	GWAS	*CYP3A4* rs17277546-A associated to metabolic trait androsterone sulfate
Rahmioglu et al. (2011)	Variability of induced CYP3A4	Classical twin study, induction with St. John’s wort	367 Twins; (99DZ, 63MZ, 43 singletons)	No	Basal activities not measured
Elens et al. (2011a)	Simvastatin-mediated cholesterol reduction	Rotterdam study subset	80 Incident simvastatin users	CYP3A4*22	*CYP3A4* rs35599367 reduced CYP3A4 activity; stronger simvastatin lipid-lowering response
Elens et al. (2011c)	Tac/CsA dose adjustment		99 Renal transplant recipients	CYP3A4*22; CYP3A5*3	Associated with altered tac/csA metabolism
Elens et al. (2011b)	Overall mean daily-dose requirement to reach the same predose tacrolimus blood concentration	International randomized controlled clinical trial (fixed dose, concentration controlled study)	185 Renal transplant recipients		*CYP3A4* rs35599367C > T associated with a significantly altered TAC metabolism and increased risk of supratherapeutic TAC concentrations early after transplantation
Zhai et al. (2011)	DHEAS levels	GWAS meta-analysis	14,846 Individuals	GWAS	*TRIM4*_rs17277546 in linkage to *3A4/5* SNPs
Elens et al. (2012)	CsA levels	Fixed dose concentration controlled study	172 *De novo* kidney transplant patients	CYP3A4*22; CYP3A5*3 CYP3A4*1B ABCB1_3435C > T	3A4*22 carriers under CsA associated with worse renal function and delayed graft function due to reduced 3A4 activity
Klein et al. (2012)	Atorvastatin AUC	Single-dose PK study	56 Healthy volunteers	CYP3A4*22 PPARa_rs4253728; ARNT_rs2134688; GR_rs258747; PGRMC2_rs3733260	CYP3A4*22 and PPARa_rs4253728 associated with higher AUC_0−∞_ ratio
Rahmioglu et al. (2012)	Variability of induced CYP3A4; measure: urinary MR quinine/3-hydroxyquinine	GWAS classical twin study, induction with St. John’s wort	310 Healthy female twins (from previous study Rahmioglu et al., 2011)	GWAS	No significant genome-wide associations to induced CYP3A4 activity; several genomic regions were highlighted that may play minor roles
Zochowska et al. (2012)	Cyclosporine A, sirolimus		100 Renal transplant recipients	CYP3A4*1B, CYP3A5*3	CYP3A5*1 and/or CYP3A4*1B carriers require significantly higher doses of cyclosporine A to reach target levels

## *CYP3A4* Polymorphisms – End of the Rope?

**Figure 1:**
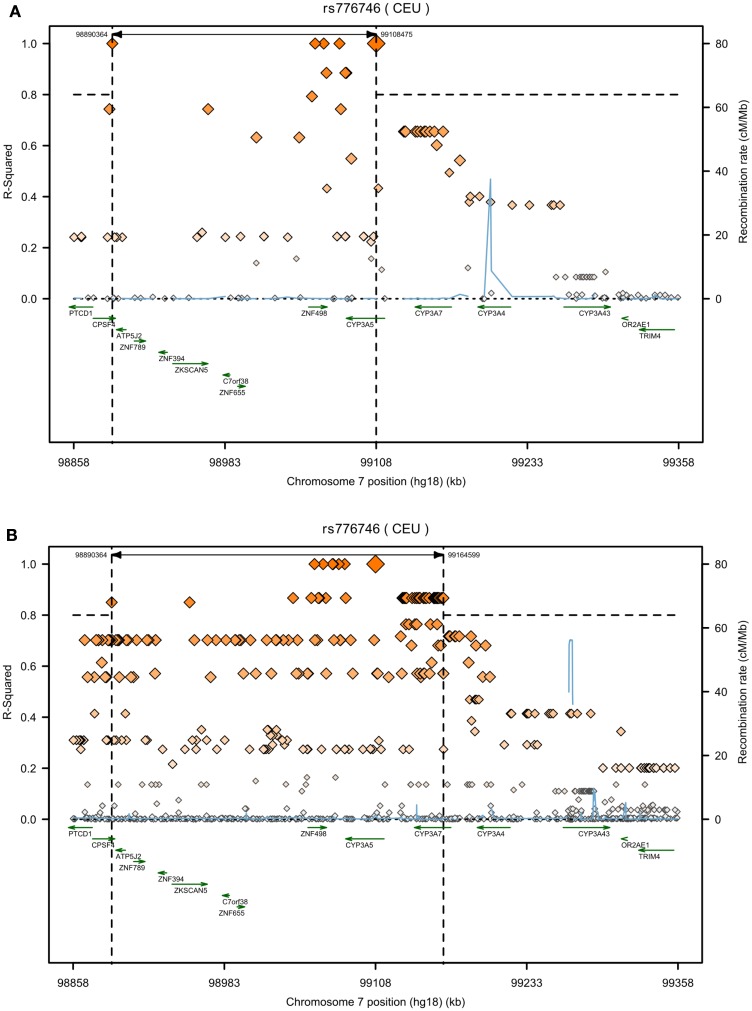
**Increase of SNP data information by the *1000 Genomes Project* within the CYP3A locus**. LD-plots are generated for rs776746 using SNAP (http://www.broadinstitute.org/mpg/snap/ldplot.php); 250 kb region up- and downstream of the target SNP; genes are marked by green arrows, SNPs are shown with their *R*^2^-square values calculated versus the target SNP rs776746 (red squares) and recombination rates are given in cM/Mb (blue line); dashed lines mark the threshold for strong linkage *R*^2^ = 0.8 (horizontal) and the corresponding region of linked SNPs (vertical); **(A)** HapMap3 Release 2, CEU **(B)** *1000 Genomes* Pilot 1, CEU.

Databases are very helpful tools to get an overview on genetic variability. However, there are major collections which serve as basis for other online browsers (Table 2). The most important collection of data concerning cytochrome P450 gene haplotypes and *in vitro* as well as *in vivo* functional impact are summarized in the *CYPallele* homepage (updated last August 31, 2011)^1^. To date besides the reference CYP3A4*1 another 21 alleles are included, comprising at least 21 amino acid changes and 2 frame shift variants. Although this collection is a useful resource for functional data related to the alleles, it only provides limited information on genetic variability. Further valuable information on *CYP3A4* SNPs and clinical pharmacogenetics is available on the homepage of *The Pharmacogenomics Knowledgebase PharmGKB*^2^. More comprehensive genetic information can be obtained from other databases, which collect SNP data of diverse projects, but in contrast to the *CYPallele* database or *PharmGKB* they lack cross reference to documented functional impact (Table 2). The dbSNP database^3^ (build 137) currently provides information on 550 SNPs for the human *CYP3A4* gene of which 21% are located within the coding region, including 61 non-synonymous amino acid changes, 18 synonymous, and 4 frameshift mutations. Remarkably, for most of the coding SNPs no MAF or reliable population related distribution data is yet available. This may soon be overcome by new data releases of the international “*1000 Genomes Project*.” This large international collaborative project aims to generate a “Deep Catalog of Human Genetic Variation” including the entire spectrum of all types of DNA changes from SNPs and small indels (insertions/deletions) to large structural variations like copy number variants and large deletions and insertions, as well as frequency information and haplotype context (The 1000 Genomes Project Consortium, 2010)^4^. Next generation sequencing technologies are used to sequence the complete diploid genomes of 2500 unidentified individuals from about 25 different populations. With the release of the Integrated Phase 1 Variant set in May 2012 phased genotype calls on 1092 samples for SNPs, short indels, and large deletions from low-coverage sequencing became available. However, no functional or medical information is being collected. The potential of the *1000 Genomes Project* for pharmacogenomics has been emphasized before (Gamazon et al., 2009). To illustrate how *1000 Genomes* data may be used in pharmacogenetics for fine analysis, e.g., in search for causal variants, Figure 1 shows exemplarily the contribution of new SNP information to the genomic region of *CYP3A* flanking the CYP3A5*3 SNP rs776746 (±250 kb). While in the *HapMap* data (Figure 1A) only eight SNPs were in strong linkage to rs776746, 49 SNPs from current *1000 Genomes* dataset were identified to be in strong LD, and interestingly also some moderately linked SNPs are located within *CYP3A4* (Figure 1B). Unfortunately, linkage information of SNPs with potential functional impact within the *CYP3A4* gene, like *1B or *22, is not available from both datasets, probably due to missing frequency data.

**Table 2:** **Overview of databases providing valuable SNP/mutation data**.

Name	link	Info^a^
Human CYPallele	http://www.cypalleles.ki.se/	Overview on functional SNPs in CYP
PharmGKB	http://www.pharmgkb.org/	Summarizing gene-drug-disease relationship, clinical PGx, PGx research, and many more; referring to dbSNP_build137
dbSNP	http://www.ncbi.nlm.nih.gov/projects/SNP/	NCBI database
*1000 Genomes Project*	http://browser.1000genomes.org/index.html	NGS project
SNPedia	http://www.snpedia.com/index.php/SNPedia	Provides information on the platforms including specific polymorphisms
MutDB (Mooney Lab)	http://www.mutdb.org/cgi-bin/mutdb.pl	Data from dbSNP (NCBI) and Swiss-Prot, includes SIFT prediction for amino acid variants
Database of genomic variation (DGV)	http://projects.tcag.ca/variation/	All SNPs from dbSNP, overview on structural genomic variations (CNV, segmental duplications/deletions, InDels)
GeneCards	http://www.genecards.org/index.shtml	dbSNP information in a compact overview with graphically illustrated frequencies
SNAP	http://www.broadinstitute.org/mpg/snap/	Displaying linkage graphically (*R*^2^ measure)

*^a^PGx, pharmacogenomics; Swiss-Prot, manually annotated and reviewed protein knowledge base at the Swiss Institute of Bioinformatics Resource portal ExPASY; NGS, next generation sequencing; SIFT, “sorting intolerant from tolerant” protein variant prediction tool*.

For the *CYP3A4* gene the current *1000 Genomes* database release (Integrated Phase 1 Variant Set; May 2012) contains in total 471 SNPs assigned to the major transcript (ENST00000336411), including NCBI dbSNP database content (build 134). The *1000 Genomes Project* to date (accessed September 2012) contributed 129 new SNPs to this collection. Besides 115 intronic variant positions 7 non-synonymous amino acid changes were previously unknown, of which four variants were reported on the *CYPallele* homepage (*4, *8, *11, *21) and only one was predicted to be deleterious by phenotype prediction tools SIFT and PolyPhen (Table 3). However, for most of the SNPs, availability of global MAF and population frequency data is still limited. Although most of the novel variants are rare, it should be noted that these can collectively make a marked contribution to the functional population variability. For individual patients, the combination of a rare deleterious variant with more frequent alleles of low function (compound heterozygosity) can be of predictive value. This was for example demonstrated for the polymorphic *CYP2B6* in a large clinical study with HIV patients under efavirenz treatment. It was shown that several individuals with elevated efavirenz plasma concentrations could be predicted when rare variants were considered together with the common low-expressor *6-allele (Rotger et al., 2007). Taking this into account, the knowledge on genetic variability of ADME (*A*bsorption *D*istribution *M*etabolism *E*xcretion) genes including *CYP3A4* in diverse populations will profit enormously from *1000 Genomes* and new candidate SNPs influencing *CYP3A4* expression and activity will certainly be discovered, although prediction or experimental testing of functional impact of the many novel variants pose a challenging task.

**Table 3:** **CYP3A4 coding SNPs provided by the *1000 Genomes Project* (selected are the SNPs for the major transcript ENST00000336411 from http://browser.1000genomes.org/Homo_sapiens/Search/Results?site=ensembl&q=cyp3a4) accessedSeptember 2012**.

ID	Chr7: bp	Alleles	Class	Source	CYPallele (activity)^a^	Type	Amino acid	AAco-ordinate^b^	SIFT	PolyPhen
rs12721634	99381661	A/G	SNP	dbSNP	*14	nsc	L/P	15 (2)	Deleterious	Probably D
rs146568511	99377652	G/A	SNP	dbSNP		nsc	P/L	43 (2)	Deleterious	Probably D
rs56324128	99375702	C/T	SNP	dbSNP	*7	nsc, ss	G/D	56 (2)	Deleterious	Probably D
rs59418896	99375666	T/C	SNP	dbSNP		nsc	Y/C	68 (2)	Deleterious	Probably D
rs3091339	99370245	T/C	SNP	dbSNP		nsc	K/E	96 (1)	Deleterious	Probably D
rs142296281	99370218	G/A	SNP	dbSNP		nsc	R/W	105 (1)	Deleterious	Probably D
rs72552799	99367788	C/T	SNP	dbSNP, 1000G	*8 (decr)	nsc	R/Q	130 (2)	Deleterious	Probably D
rs4987161	99366081	A/G	SNP	dbSNP	*17 (decr)	nsc	F/S	189 (2)	Deleterious	Probably D
rs139541290	99366075	A/G	SNP	dbSNP		nsc	V/A	191 (2)	Deleterious	Probably D
rs55901263	99365994	G/C	SNP	dbSNP	*5	nsc	P/R	218 (2)	Deleterious	Probably D
rs75726589	99364854	A/G	SNP	dbSNP		nsc	L/P	233 (2)	Deleterious	Probably D
rs190354371	99361563	A/G	SNP	1000G		nsc	L/P	314 (2)	Deleterious	Probably D
1000GENOMES_ 7_99361548	99361548	T/C	SNP	1000G	*21	nsc	Y/C	319 (2)	Deleterious	Probably D
rs71581998	99359841	A/T	SNP	dbSNP		nsc	V/E	359 (2)	Deleterious	Probably D
rs67784355	99359829	G/A	SNP	dbSNP, 1000G	*11 (decr)	nsc	T/M	363 (2)	Deleterious	Probably D
rs113716682	99359715	A/G	SNP	dbSNP		nsc	L/P	401 (2)	Deleterious	Probably D
rs143966082	99359710	G/A	SNP	dbSNP		nsc	R/C	403 (1)	Deleterious	Probably D
rs72552797	99359685	G/A	SNP	dbSNP		nsc	P/L	411 (2)	Deleterious	Probably D
rs4986909	99359670	G/A	SNP	dbSNP	*13 (decr)	nsc	P/L	416 (2)	Deleterious	Probably D
rs4986910	99358524	A/G	SNP	dbSNP	*3	nsc	M/T	445 (2)	Deleterious	Probably D
rs72552796	99358521	C/T	SNP	dbSNP		nsc	R/K	446 (2)	Deleterious	Probably D
rs71583803	99358470	A/C	SNP	dbSNP		nsc	F/C	463 (2)	Deleterious	Probably D
rs78764657	99377692	G/C	SNP	dbSNP		nsc	H/D	30 (1)	Deleterious	Possibly D
rs140422742	99375669	T/C	SNP	dbSNP		nsc	K/R	67 (2)	Deleterious	Possibly D
rs57409622	99367428	G/A	SNP	dbSNP		nsc	R/W	162 (1)	Deleterious	Possibly D
rs71581996	99361591	C/A	SNP	dbSNP		nsc	A/S	305 (1)	Deleterious	Possibly D
rs188389063	99381698	G/C	SNP	1000G		nsc	L/V	3 (1)	Deleterious	Benign
1000GENOMES_ 7_99381694	99381694	A/G	SNP	1000G		nsc	I/T	4 (2)	Deleterious	Benign
COSM42988	99381647	C/T	Somatic_SNV	COSMIC		nsc	V/M	20 (1)	Deleterious	Benign
rs145582851	99364062	C/T	SNP	dbSNP		nsc	R/Q	268 (2)	Deleterious	Benign
rs148633152	99359730	A/G	SNP	dbSNP		nsc	I/T	396 (2)	Deleterious	Benign
rs149870259	99358596	T/C	SNP	dbSNP		nsc	K/R	421 (2)	Deleterious	Benign
rs28371760	99358498	A/-	del	dbSNP		nsc, fs	L/I	454 (1)	Deleterious	Benign
rs150559030	99358488	A/T	SNP	dbSNP		nsc	I/N	457 (2)	Deleterious	Benign
rs12721627	99366093	G/C	SNP	dbSNP	*16 (decr)	nsc	T/S	185 (2)	Tolerated	Possibly D
rs55785340	99365983	A/G	SNP	dbSNP	*2	nsc	S/P	222 (1)	Tolerated	Possibly D
COSM35658	99381689	C/T	Somatic_SNV	COSMIC		nsc	D/N	6 (1)	Tolerated	Benign
rs140355261	99381687	G/C	SNP	dbSNP		nsc	D/E	6 (3)	Tolerated	Benign
COSM42989	99381680	T/C	Somatic_SNV	COSMIC		nsc	M/V	9 (1)	Tolerated	Benign
rs55951658	99367825	T/C	SNP	dbSNP, 1000G	*4	nsc	I/V	118 (1)	Tolerated	Benign
rs147752776	99367818	A/G	SNP	dbSNP		nsc	I/T	120 (2)	Tolerated	Benign
rs4986907	99367427	C/T	SNP	dbSNP	*15	nsc	R/Q	162 (2)	Tolerated	Benign
rs72552798	99367404	C/T	SNP	dbSNP	*9	nsc	V/I	170 (1)	Tolerated	Benign
rs3208361	99366070	T/C	SNP	dbSNP		nsc	I/V	193 (1)	Tolerated	Benign
rs113667357	99366047	T/A/C	SNP	dbSNP		nsc	Q/H	200 (3)	Tolerated	Benign
rs181612501	99365992	A/G	SNP	1000G		nsc	F/L	219 (1)	Tolerated	Benign
rs3208363	99364798	A/C	SNP	dbSNP		nsc	S/A	252 (1)	Tolerated	Benign
1000GENOMES_ 7_99364768	99364768	C/T	SNP	1000G		nsc	E/K	262 (1)	Tolerated	Benign
rs28371759	99361626	A/G	SNP	dbSNP	*18	nsc	L/P	293 (2)	Tolerated	Benign
rs138675831	99361618	C/T	SNP	dbSNP		nsc	V/M	296 (1)	Tolerated	Benign
1000GENOMES_ 7_99361606	99361606	T/C	SNP	1000G		nsc	I/V	300 (1)	Tolerated	Benign
rs10250778	99359871	G/T	SNP	dbSNP		nsc	T/N	349 (2)	Tolerated	Benign
rs145669559	99359812	T/C	SNP	dbSNP		nsc	I/V	369 (1)	Tolerated	Benign
rs12721629	99359800	G/A	SNP	dbSNP	*12 (decr?)	nsc	L/F	373 (1)	Tolerated	Benign
rs142425279	99359734	T/C	SNP	dbSNP		nsc	M/V	395 (1)	Tolerated	Benign
rs139109027	99358581	T/C	SNP	dbSNP		nsc	N/S	426 (2)	Tolerated	Benign
rs1041988	99358566	A/G	SNP	dbSNP		nsc	I/T	431 (2)	Tolerated	Benign
rs4986913	99358459	G/A	SNP	dbSNP	*19	nsc	P/S	467 (1)	Tolerated	Benign
rs181210913	99358450	C/T	SNP	1000G		nsc	E/K	470 (1)	Tolerated	Benign
rs138105638	99364063	G/A	SNP	dbSNP		Stop	R/*	268 (1)	–	–
rs34784390	99364036– 99364035	−/T	ins	dbSNP		fs		277 (2)	–	–
rs4646438	99364035– 99364034	−/T	ins	dbSNP	*6	fs		277 (3)	–	–
rs72552795	99358466– 99358465	−/G	ins	dbSNP		fs		465 (1)	–	–
rs67666821	99355807– 99355806	−/T	ins	dbSNP	*20 (no)	fs		488 (1)	–	–

*^a^Derived from http://www.cypalleles.ki.se/; Information not provided by *1000 Genomes**.

*^b^Number corresponds to the codon, variant nucleotide of the triplet is given brackets; nsc, non-synonymous coding; fs, frameshift; D, damaging*.

## Genes Influencing CYP3A4 Phenotype Outside the *CYP3A* Locus

**Figure 2:**
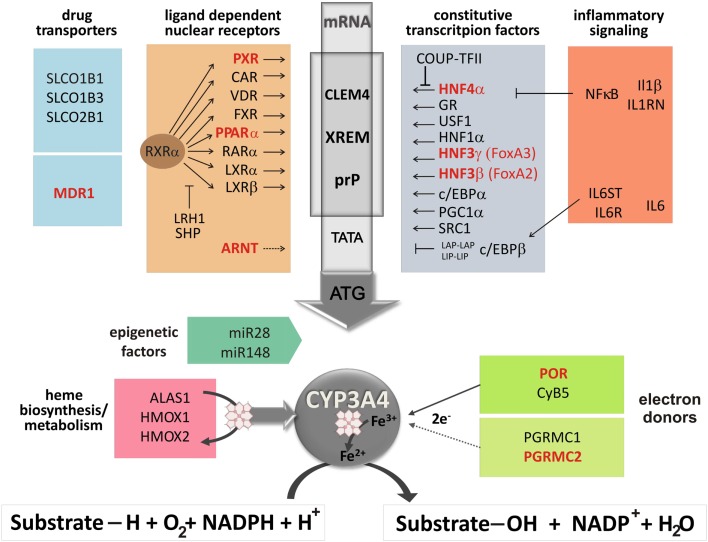
**Regulation network of CYP3A4 phenotype expression adapted from Klein et al. (2012)**. The scheme displays the promoter region of *CYP3A4* covering constitutive liver enhancer module CLEM4, xenobiotic responsive enhancer module XREM, and the proximal promoter prP. Genes of the constitutive and inducible pathways (Jover et al., 2002; Burk and Wojnowski, 2004) drug transporters, inflammatory signaling (Martínez-Jiménez et al., 2005; Aitken et al., 2006), possible miRNA interference (Pan et al., 2009), and of the heme biosynthesis/metabolism pathway as well as potential electron donors are listed. Genes with documented influential polymorphisms on CYP3A4 expression are depicted in red. Abbreviations: ALAS1, δ-aminolevulinate-synthase; ARNT, aryl hydrocarbon receptor nuclear translocator; CAR, constitutive androstane receptor; c/EBP, CCAAT/enhancer binding protein-α; Coup-TFII, COUP transcription factor II; CyB5, cytochrome B5; CYP3A4, cytochrome P450 3A4; FXR, farnesoid X-receptor; GR, glucocorticoid receptor; HMOX, heme-oxygenase; HNF1α, hepatocyte nuclear factor 1 homeobox A; FoxA2 (HNF3β), forkhead box A2; FoxA3 (HNF3Γ), forkhead box A3; HNF4α, hepatocyte nuclear factor 4 α; IL1β, interleukin-1 β; IL1RN, interleukin-1 receptor antagonist; IL6, interleukin-6; IL6R, interleukin-6 receptor; IL6ST, interleukin-6 signal transducer; LRH1, liver receptor homolog 1; LXRα/β, liver X-receptor; MDR, multi-drug resistance protein; NF-κB, nuclear factor-κB subunits; PGRMC, progesterone receptor membrane component; POR, P450 oxidoreductase; PPARα, peroxisome proliferator-activated receptor α; RARα, retinoic acid receptor; RXRα, retinoid X-receptor; PXR, pregnane X-receptor; SHP, short heterodimer partner; SLCO, solute carrier organic anion transporters; USF-1, upstream stimulatory factor 1; VDR, vitamin D receptor.

Regulation of CYP3A4 phenotype expression is enormously complex including influences from networks of nuclear receptors and other transcription factors (Pascussi et al., 2008), hormonal and inflammatory pathways (Aitken et al., 2006), heme and protein synthesis, and degradation pathways, as well as components of monooxygenase complexes and their interaction partners (see in Figure 2). Potentially influential polymorphisms in these genes on *CYP3A4* expression have been studied only in recent years by single gene or pathway-directed approaches.

NADPH:cytochrome P450 oxidoreductase (POR) is a microsomal flavoprotein and an obligatory electron donator in the microsomal P450 monooxygenase reaction. In contrast to the multiplicity of CYPs, mammals have only a single *POR* gene. In humans the gene is located on chromosome 7q11.2 and spans about 72 kb, coding for a 680 amino acid protein. Complete deletion of the *por* gene in mouse is embryonically lethal most likely due to deficient adrenocortical steroidogenesis (Shen et al., 2002; Otto et al., 2003). In contrast, liver-specific *por* knockout leads to phenotypically and reproductively normal mice that accumulate hepatic lipids and have a drastically diminished capacity for hepatic drug metabolism (Gu et al., 2003; Finn et al., 2007).

The amount of POR in human liver is stoichiometrically ∼5- to 10-fold lower compared to the microsomal CYP pool, and hepatic POR levels are correlated to several P450 monooxygenase activities, suggesting that it represents a limiting factor (Huang et al., 2008a; Gomes et al., 2009). Functional polymorphisms in *POR* should thus be expected to influence CYP activity. In recent years rare POR missense mutations in humans were discovered that impair POR function and cause disordered steroidogenesis, ambiguous genitalia, and Antley–Bixler syndrome (Flück et al., 2004; Flück and Pandey, 2011). The *CYPalleles* website currently lists 41 distinct star-alleles, most of which represent very rare mutations, but common polymorphisms also exist (Huang et al., 2005, 2008a).

In particular the A503V variation (POR*28) is common with frequencies ranging from 19 to 37% in all major ethnicities. In recombinant systems the variant retained >50% of the wild type activity toward several CYPs (Huang et al., 2008b; Sandee et al., 2010) and CYP3A4 activity was influenced in a substrate-dependent way (Agrawal et al., 2010). In an *in vivo* study it was found that POR*28 TT genotype was associated with a 1.6-fold increase in CYP3A midazolam 1′-hydroxylase activity compared with POR*28 C carriers, a finding that could be replicated in an independent cohort (Oneda et al., 2009). Similarly, in a cohort of allograft recipients under tacrolimus therapy, POR*28 T-allele carriers had significantly higher tacrolimus dose requirements compared to non-carriers but only if they were genotypic CYP3A5 expressors (i.e., presence of at least one CYP3A5*1 allele; De Jonge et al., 2011). Thus, the effect of this common *POR* variant appears to depend on the CYP isozyme as well as the substrate.

Numerous additional genetic *POR* variants have been identified (Hart et al., 2008; Huang et al., 2008a). With regard to CYP3A4, several variants were described to affect its function in liver, *in vitro* or *in vivo*. A multivariate analysis in human liver microsomes identified three intronic *POR* variants that affected several CYP activities and that accounted for 60% of the microsomal atorvastatin hydroxylase variability, in concert with the donor’s sex (Gomes et al., 2009). Chen et al. analyzed six full length protein variants for their impact on CYP3A4-mediated testosterone hydroxylation and on CYP2B6-mediated bupropion hydroxylation in a recombinant system. Several *POR* variants showed markedly differential effects on both activities (Chen et al., 2012). *In vivo*, reduction in hepatic drug metabolizing CYP3A4 activities caused by *POR* mutations was also described in patients with disordered steroid metabolism (Flück et al., 2010). Taken together these recent advances indicate that *POR* variants are a complex but potentially relevant source of genetic variation for CYP3A-related drug oxidation phenotypes (Miller et al., 2011).

The xenosensors pregnane X-receptor (PXR, *NR1I2*) and constitutive androstane receptor (CAR, *NR1I3*) are important transcription factors in the regulatory network of *CYP3A4* constitutive and inducible expression. Both genes are polymorphic and genetic variants have been studied for impact on *CYP3A4* transcription (Lamba et al., 2005). Despite existence of numerous alternative transcripts, naturally occurring functional polymorphisms of *CAR* that contribute to CYP3A4 phenotypic expression are not well documented. In contrast, the functional impact of genetic *PXR* variants has been studied more thoroughly. For example, the protein variants V140M, D163G, and A370T, discovered in an exon sequence screening of *PXR* gene in DNA samples of several ethnicities, were shown to exhibit altered basal or induced transactivation activity of *CYP3A4* promoter using recombinant variant expression and promoter-reporter constructs (Hustert et al., 2001b). Resequencing analysis of 46 liver tissue samples of Caucasian origin and hepatocytes identified SNPs in the *PXR* promoter and intron 1 associated with CYP3A4 mRNA expression, mainly due to destruction of putative transcription factor binding sites within the regulatory region of *PXR* by polymorphisms in strong linkage (Lamba et al., 2008). Moreover, the PXR*1B haplotype, which is tagged by the SNPs 2654T > C and IVS6-17C > T, was related to doxorubicin clearance in Asian breast cancer patients (Sandanaraj et al., 2008). Further studies analyzing patient cohorts under fosamprenavir/lopinavir or artemisinin treatment gave additional hints to some *PXR* variants that may affect inducibility of CYP3A4 phenotype expression (Svärd et al., 2010; Piedade et al., 2012). The occurrence of two SNPs in the 3′UTR region of *PXR*, rs3732359 and rs3732360, has been shown to be associated with altered midazolam 1′-hydroxylation in a liver bank and with more pronounced effect in midazolam clearance of healthy volunteers of African American descent (Oleson et al., 2010). Two recent studies included *PXR* polymorphisms in their pathway targeted pharmacogenetic approach on variable CYP3A4 expression in liver samples (Lamba et al., 2010; Klein et al., 2012). Although in the former study *PXR* rs1523130 was significantly associated with *CYP3A4* mRNA expression by univariate analysis and in the multivariate model, the latter study did not document a significant contribution of *PXR* SNPs in univariate as well as in multivariate statistical models on mRNA, protein or activity. In summary, contribution of *PXR* variants to CYP3A4 expression remains controversial and may be limited and dependent on the population studied.

Based on the observation that CYP3A4 and the multi-drug resistance protein P-glycoprotein [P-gp, product of the *MDR1 (ABCB1)* gene] display largely overlapping substrate selectivity (Wacher et al., 1995; Patel and Mitra, 2001; Pal and Mitra, 2006), it has been hypothesized that changes in P-gp expression may influence the intracellular concentration of endogenous or exogenous substances that potentially lead to *CYP3A4* induction, thus contributing to CYP3A4 expression differences (Lamba et al., 2006). Although data supporting this hypothesis have been reported in one human liver association study (Lamba et al., 2010), they were not reproduced in another study (Klein et al., 2012).

In search for additional influential genes, Lamba et al. (2010) phenotyped 128 livers by quantitative real-time PCR for expression of *CYP3A* genes and identified a functional CCT-repeat polymorphism in the *FoxA2* (*HNF3*α) gene to be associated with higher expression of FoxA2 mRNA and its target genes *PXR* and *CYP3A4*. Polymorphisms in *FoxA2*, *HNF4*α, *FoxA3* (*HNF3*Γ), *PXR*, *MDR1*, and the *CYP3A4* promoter together with sex explained 24.6% of the variation in hepatic CYP3A4 mRNA expression. However the study lacked information on the relevance of these variations for CYP3A4 protein and activity.

More recently, we carried out extensive candidate gene approaches on 150 Caucasian liver samples phenotyped for CYP1A2 and CYP3A4 mRNA and protein levels, as well as enzyme activity (Klein et al., 2010, 2012). With respect to CYP3A4, we identified SNPs in the Ah-receptor nuclear translocator (*ARNT*), glucocorticoid receptor (*GR*), progesterone receptor membrane component 2 (*PGRMC2*), and peroxisome proliferator-activated receptor alpha (*PPARA*) to be consistently associated with CYP3A4 phenotype in human liver in the multivariate analysis. Validation in an atorvastatin-treated volunteer cohort confirmed decreased atorvastatin-2-hydroxylation in carriers of *PPARA* SNP rs4253728. Moreover, homozygous carriers of the variant had reduced PPARα protein expression in liver, and shRNA-mediated *PPARA* gene knock-down in primary human hepatocytes decreased mRNA expression levels of *CYP3A4* by more than 50%. Multivariate analysis revealed that two linked *PPARA* SNPs alone explained ∼8–9% of the atorvastatin hydroxylase activity variation, whereas all genetic and non-genetic factors together accounted for ∼33% of atorvastatin 2-hydroxylase variation in this liver cohort (Klein et al., 2012). This result was somewhat unexpected, because PPARα had so far not been considered as a major regulator of *CYP3A4*, although inducing effects by fibrates had been noted before in human, but not mouse primary hepatocytes (Prueksaritanont et al., 2005; Rakhshandehroo et al., 2009). The interesting question of course is, whether *CYP3A4* is a direct target of PPARα or whether indirect regulation, e.g., via nuclear receptor crosstalk resulting in downregulation of PXR (Aouabdi et al., 2006; Takagi et al., 2008) is the causative mechanism. Recent data from our group suggest, however, that PPARα indeed binds to several non-consensus PPARα-response elements (PPREs) in the *CYP3A4* promoter to activate transcription in a ligand-dependent manner (Thomas et al., 2013). Because PPARα is a master regulator of lipid homeostasis and energy balance, these results indicate a novel connection between endogenous and xenobiotic metabolism.

## New Aspects/Outlook

Several genome-wide association studies with a total number of over 1000 human liver samples identified a large number of novel expression quantitative trait loci (eQTL) for numerous liver-expressed genes (Schadt et al., 2008; Yang et al., 2010; Innocenti et al., 2011; Schröder et al., 2013). Although the advantage of this approach is the unbiased way of investigation, results were rather disappointing with respect to CYPs, probably due to lack of statistical power. Thus, mainly polymorphisms in *CYP2D6* and *CYP3A5* were reproduced in these studies (Schadt et al., 2008; Schröder et al., 2013). Building up on one eQTL study, Yang et al. used the genome-wide SNP data of 466 livers to search for associations with enzyme activities determined for 9 CYPs (i.e., activity- or aQTLs). A total of 54 SNPs influencing 8 CYP activities were identified, of which 30 influenced CYP2D6 in *cis* (i.e., SNPs were located within ±1 Mb of the *CYP2D6* gene), whereas all remaining 24 SNPs were described as *trans*-acting elements, and only one of these (rs12041966 located on chromosome 1) influenced testosterone hydroxylation (Yang et al., 2010). Unfortunately, functional annotation of the *trans*-acting SNPs was not possible in this study, and these results thus await further experimental confirmation.

A further level of potential importance, which has not yet been explored in terms of genetic variability concerns the influence of epigenetic processes on pharmacologically relevant genes and drug response (Gomez and Ingelman-Sundberg, 2009). SNPs in DNA methylation regions or miRNAs and miRNA binding sites, as well as miRNA copy number variations may influence target gene expression (Schmeier et al., 2011). Thus, CYP3A4 expression was shown to be directly regulated by miRNAs but can also be influenced indirectly by miRNA regulation of transcriptional regulators such as PXR and VDR (Pan et al., 2009). However, polymorphic variation within this regulatory pathway has not yet been analyzed. Furthermore, additionally to regulatory polymorphisms (rSNPs) affecting transcription, structural RNA polymorphisms (termed srSNPs) are suggested to influence RNA function (splicing, turnover, translation) and to display promising biomarkers (Sadee et al., 2011; Lee et al., 2012).

The *ENCODE* project (*ENC*yclopedia *O*f *D*NA *E*lements)^5^ systematically analyzes functional DNA elements in the human genome (e.g., binding data for more than 100 transcription factors, DNAse sensitive sites, methylation, chromatin interaction, and genotyping from multiple cell types) and may provide new hypotheses on functional consequences of SNPs located especially in non-coding DNA-regions (Bernstein et al., 2012; Gerstein et al., 2012; Yip et al., 2012).

In conclusion, the predictive power of currently known genetic polymorphisms with relevance for CYP3A4 *in vivo* phenotype is still far away from the expected 60–80% of genetic determination. The use of next generation sequencing approaches for the identification of causal variants in *CYP3A4* as well as the numerous genes of its many influential pathways may lead to the identification of many more rare and common DNA variants that together account for a sizeable fraction of this variability. The examples of *POR* and *PPARA* demonstrate that CYP3A4 variability is at least in part determined by polymorphisms in genes outside the *CYP3A4* locus. However, investigating and validating *trans*-acting factors is much more difficult due to the more indirect nature of interaction which is more likely to be masked by covariates, thus necessitating larger studies, *in vitro* or *in vivo*. In addition, mathematical algorithms are needed to combine many genetic variants, some of which contribute only small fractions to the total variability, into practically useful signatures for application on clinical studies and individualized medicine.

## Conflict of Interest Statement

The authors declare that the research was conducted in the absence of any commercial or financial relationships that could be construed as a potential conflict of interest.

## Footnotes

^1^http://www.cypalleles.ki.se/

^2^http://www.pharmgkb.org/

^3^http://www.ncbi.nlm.nih.gov/projects/SNP

^4^http://www.1000genomes.org

^5^http://genome.ucsc.edu/ENCODE/
